# The Potential Uses of Baobab Tree's Medicinal Effects in Dentistry: A Literature Review

**DOI:** 10.7759/cureus.49304

**Published:** 2023-11-23

**Authors:** Jameel A Abuljadayel

**Affiliations:** 1 Preventive Dentistry, Umm Al-Qura University, Makkah, SAU

**Keywords:** anti-inflammatory, antioxidants, dentistry, adansonia digitata, dental biomaterials, baobab

## Abstract

Adansonia digitata (Baobab) tree is an African tree with a long history in traditional medicine. The local inhabitants of Africa have been using the different tree components to treat medical diseases, such as fever, diarrhea, malaria, cough, dysentery, and microbial infections. Recently, the tree gained the attention of scientists due to its medical and pharmaceutical properties and nutritional values, which generated a myriad number of investigations regarding its phytochemical and macro- and micronutrient contents. The fruit pulp is especially rich in vitamin C, pectin, fibers, and minerals such as calcium, magnesium, potassium, phosphorus, zinc, iron, and copper. Additionally, the leaves contain high levels of calcium, while the seeds are considered a good source of protein and fat. Altogether, they contain a variety of polyphenols, fatty acids, and amino acids. The tree extracts possess potent antioxidant, cell-protective, and anti-inflammatory activities. However, no information was found in the literature about the use of Baobab tree products in the dental field. The aim of this review is to discuss the well-documented medical effects and chemical and mineral components of the different Baobab tree parts from a dental point of view to open more areas of research concerning its potential applications in the dental field. Antioxidants and vitamin C are known to help in maintaining healthy periodontal and gingival tissues. They also help in wound healing and alveolar bone integrity. Moreover, phytochemicals and phenolic compounds have been utilized in controlling dental plaque and manufacturing intracanal medications as they manifest antimicrobial and anti-inflammatory activities. Furthermore, calcium and phosphorus incorporation in dental biomaterials is commonly used in vital pulp therapy and repairing bone defects. After reviewing the reported medicinal and pharmaceutical activities of the Baobab tree, it can be inferred that the tree extracts possess potential uses in the dental field, which requires further investigation for validation.

## Introduction and background

Adansonia digitata (AD/Baobab) is a native fruit that grows and originates in the sub-Sahara areas of Africa [1,2]. For example, it can be initially found in Sudan, Nigeria, Mali, Burkina Faso, Senegal, Kenya, Mozambique, etc. [1-6]. The tree itself is a part of the Malvaceae family, and it has an iconic shape and various common names, such as the upside-down tree, the monkey-bread tree, the small pharmacy tree, and the chemist tree [1,7].

The native population of Africa used the different parts of the tree throughout history in their diet and traditional medicine [1,2,5-8]. The whole components of the tree (fruit, leaves, seeds, and cortex) have been traditionally used as a medicine for several diseases, such as fever, diarrhea, malaria, cough, dysentery, hemoptysis, tuberculosis, microbial infections, and worms [2,4,5,7], while the seeds and oil are used to treat muscle wounds, dandruff, and other skin diseases [7,9]. These therapeutic uses were justified lately in the literature and linked to the immunostimulant, antioxidant, analgesic, antiviral, antimicrobial, anticarcinogenic, anti-depressive, hepatoprotective, cardioprotective, antidiabetic, and anti-inflammatory potentials of the tree's components [2,5,7,10-20].

Many reports have analyzed the chemical composition of the different tree components [1,2,7,8,21,22]. These components' medical and pharmaceutical effects are evident in the literature [23-27]. However, the literature needs more information about using Baobab tree products in the dental field. Thus, this review aims to discuss the published medical bioactive benefits of the various Baobab tree components from a dental point of view and then suggests the potential utilization areas of these extracts among the different dentistry disciplines.

## Review

Mineral contents and chemical composition of the AD tree

The Baobab tree is believed to be rich in chemical compounds, vitamins, nutrients, and minerals, which possess pharmacological, medicinal, and bioactive features [2,4,5,7]. Regarding the mineral contents, reports have shown that the fruit pulp contains high amounts of Ca, Mg, K, P, Zn, Fe, and Cu [28-30]. Chadare et al. have comprehensively reviewed the composition of the different parts of the AD tree and reported that the average calcium contents in the leaves, fruit pulp, and seeds were 1,582.3 mg/100 g, 301.8 mg/100 g, and 252 mg/100 g, respectively [8]. The same review has shown that phosphorus average levels were 453 mg/100 g, 274 mg/100 g, and 106 mg/100 g in the seeds, leaves, and pulp, respectively [8]. In an in vitro study that measured the mineral contents of the root tubers of the Baobab tree, calcium was shown to be the highest available mineral (42.91 mg/100 g) [1]. Additionally, Evang et al. showed that calcium content in the Baobab fruit pulp is equal to 408 mg/100 g in their sample [28]. Another investigation has completely replaced the skimmed milk in their ice cream contents with the pulp flour of the Baobab fruit and still got a considerable amount of calcium. In contrast, phosphorus has directly increased with the consequent increase of the Baobab fruit pulp percentages as an ingredient [30]. In addition, a published report has investigated the calcium and phosphorus amounts in cake manufacturing after the partial replacement of wheat flour with Baobab fruit pulp flour and reported a significant increase of both elements levels when the Baobab fruit pulp is incorporated [29].

Regarding vitamin C, many reports have shown that Baobab fruit pulp is tremendously rich in vitamin C [10,28,31]. It was reported that the average level of vitamin C in the pulp of AD is up to 290 mg/100 g [8], which is considered up to 10 times higher than vitamin C levels in orange pulp and significantly higher than in other different fruits [2,7]. In other words, consuming 20 g of the Baobab pulp would cover 140%-240% and up to 40%-70% of the recommended daily intake for a child and pregnant women, respectively [8].

Chemically, the Baobab tree components (fruit pulp, fruit shell, leaves, seeds, seed oil, stem bark) were thoroughly investigated and shown to be extremely rich in phytochemicals and polyphenols, such as hydroxybenzoic acid, protocatechuic acid, dihydrocaffeic acid, hydroxycinnamic acid, gallic acid, catechin, flavonoids, saponin derivatives, and terpenoids [21,23-26]. Additionally, fatty acids such as linoleic, palmitic, and oleic acids were found in tree extracts [21,25]. Investigations have also shown that considerable amounts of amino acids were found, such as methionine, L. arginine, lysine, and proline [8,10,18,23-27,29,30,32-37]. With such components, it is no wonder that the tree has been known as "the chemist tree” as these compounds possess countless medicinal and pharmaceutical benefits.

Potent and bioactive compounds of AD and their medicinal activities

All AD tree components are traditionally widely known for their medicinal potency [1]. Despite that, the reported level of activity is affected by several factors, such as the area and the way of harvesting, the storage process, the age of samples, the soil type, genes, and the methodology of analysis [8]. However, there is a consensus in the literature that Baobab/AD possesses a high level of antioxidant activity [4,5,7,8,11,12,14,17,22,26]. Antioxidant activity was reported concerning every part of the Baobab tree component (fruit pulp, shell, leaves, seeds, and stem bark), and it was linked to the phytochemical, polyphenolic, and vitamin C contents of the tree components [10,24,26,35]. It was reported that the integral antioxidant capacity of the pulp is up to 100 times higher than that of the orange pulp [8]. Moreover, Baobab leaves show higher antioxidant capacity than other widely known fruits for their antioxidant activities, such as kiwi, strawberries, and apples [8]. Recent animal studies showed the protective effect of Baobab pulp extracts through their potent antioxidant activity against testicular damage induced by cotton seed extract and cadmium chloride in Wistar rats [33,34]. Additionally, another study showed that by suppressing oxidative stress, aqueous Baobab leaf extract reduced the cortical neurodegeneration caused by lead acetate in rats [27]. Baobab pulp has been shown to have a significant cardioprotective effect when used as a treatment for isoproterenol-induced myocardial oxidative stress in experimental rats, as it was able to reduce oxidative stress and blocks the increase of cardiac marker enzymes, which in turn reduces the extent of myocardial damage [14].

The antidiabetic, anti-inflammatory, and anti-dyslipidemia potency of Baobab tree extracts were also reported in the literature [10,13,18,23]. It was shown that Baobab can reduce the blood sugar response to meals high in carbohydrates both in vitro and in vivo [13]. Furthermore, the antidiabetic action of Baobab fruit extracts was tested in a model of diabetic rats, and results showed that they significantly improved lipid and glucose metabolism in addition to exerting an antioxidant effect [24]. Another animal study has shown that the fruit pulp of AD exhibited hypoglycemic, anti-inflammatory, and antilipidemic properties, which were reflected in a lower plasma atherogenic index [10]. Furthermore, it restored the stability of hepatic and renal biomarkers and acted as a restoring agent on the pathological damage of the tissues of the heart, liver, and kidneys [10]. Another in vivo study has examined the effect of incorporating 30 mg of Baobab fruit pulp powder into the medication protocol of dyslipidemia patients and found a significant reduction in total cholesterol and triglycerides after four weeks in comparison with the control group who were on medication only [38]. It is worth mentioning that the mechanisms of all these medicinal effects are not yet fully understood; however, they are linked synergistically to the antioxidant and anti-inflammatory potency of the Baobab fruit [10]. Findings of common AD compounds, elements, and minerals are summarized along with their medical effect in Table 1.

**Table 1:** The most prominent compounds of AD and their medicinal impact.

Part of the tree	Elements or compounds	Reported medicinal effects
Fruit	Phytochemicals	Hepatic and renoprotective [10,25].
	Phenolic compounds	Antioxidant [10,26,30,33,34,39,40], hypoglycemic and antidiabetic effects [13,24,39-41]. Also enhances cognitive performance [31,42].
	Vitamin C, Ca, and Mg	Anti-inflammatory effect (vitamin C) [10], and potentially improve anemia cases [28].
	Amino acids (e.g., methionine, L. arginine, lysine, proline)	Building up protein, antioxidants, inducing collagen and elastin, and enhancing vascular integrity [10].
	Pectin fibres	Anti-Hyper cholesterol [24,38].
Fruit shell	Phenolic compounds	Antidiabetic and antioxidant [23].
Seeds oil	Phenolic compounds	Antioxidant and cell protective [43].
	Fatty acids (e.g., linoleic, palmitic, and oleic acid)	Hydrating, moistening, and non-irritant to the skin [44].
Leaves	Phytochemicals	Anthelmintic activity [37].
	Phenolic compound	Neuroprotective and antioxidant effect [27,32]. Antihypertensive, Anti-dyslipidemia, and Anti-metabolic syndrome effects [18]. Hepatoprotective [32].
Stem bark	Phenolic compounds	Antihypertensive, Antioxidant, Cardiovascular Protective and Hepato-protective [35,36].

Potential uses of AD (Baobab) extracts in dentistry according to its published compounds

On a cellular level, the oxidation process will form free radicals, which in turn initiate destructive reactions in human cells [45]. Antioxidant compounds are responsible for inhibiting oxidation reactions, as they are adapted to halt free radicals before they attack human cells [45]. The antioxidant effect of Baobab tree components is well-documented in the literature, as previously discussed. This feature would be critical in dentistry and dental medicine, as cell oxidation can occur in many oral, gingival, and periodontal diseases. In a recent systematic review with meta-analysis, Castro et al. have shown that using antioxidants as an adjunctive treatment with non-surgical periodontal therapy would significantly help control the periodontal status in patients with periodontitis [46]. Moreover, Woelber et al. demonstrated that rich antioxidants in dietary habits could significantly decrease gingivitis [47]. In addition, Alpan et al. have tested a potent antioxidant (taxifolin) and found that it can decrease cell death and improve the alveolar bone formation in their periodontitis rats model [48]. 

Having both potent antioxidant activity and high levels of vitamin C suggests more dental applications of the Baobab extracts. Several in vivo and in vitro studies [49-55] have suggested that vitamin C utilization would greatly benefit tissue, wound, and bone healing after surgical procedures and implant placement. Moreover, a recent investigation has revealed a novel potential of vitamin C as an anti-cariogenic agent [56].

Phytochemicals and phenolic compounds are widely used in dentistry as they are known for their antiseptic, antibacterial, analgesic, and anti-inflammatory properties [57]. They were reported to be incorporated in the mouthwash and toothpaste industry, as they show plaque control abilities [58-60], and potency against cariogenic bacteria [61-64]. Additionally, phenol and phenolic derivatives are used as intracanal medications and pulp therapy medicaments [65-67], impression disinfectants [68,69], and in pain control and anesthesia [57]. In addition, it was reported that polyphenols would improve the biological and mechanical stability of dentine structure and matrix [70], which would enhance the restorative and adhesive properties of dental materials.

Fatty acids are known as components that might exist as a part of a molecule or act separately in the body, and the body organisms utilize them for metabolic operations in general and physical cell growth [71]. Regarding dentistry, several reports have shown that fatty acids have a role in improving periodontal health by reducing inflammatory mediators, decreasing clinical attachment loss, diminishing bone resorption [72-75], and exhibiting an antibacterial effect [76]. On the other hand, some fatty acids in the gingival fluids could be used as a biomarker to determine the periodontal health status and diagnose periodontal diseases [77,78].

Amino acids are known to play an essential role as the building blocks of protein in the human body; they are also needed to maintain good health and function of body organs [79]. It was reported that amino acids correlate with caries experience in humans. Masoudi et al. have shown that amino acid concentrations in saliva could be used as a biomarker for caries [80]. Moreover, it was reported that different amino acids are utilized to fabricate chemical caries removal products, such as Caridex and Carisolv [81]. Another application of amino acids was investigated, as it has been reported that novel dental restorative materials and cements were made by incorporating different amino acids [82-85]. Zilm et al. showed that D-amino acids can reduce the bacterial biofilm of Enterococcus faecalis in the presence of other antibacterial agents in an in vitro model [86].

Regarding calcium, phosphorus, and dentistry, a very long list of applications could be made as these minerals have an essential role in teeth and bone formation [79]. The tooth's chemical structure (enamel and dentin) is mainly composed of Ca and P [87]. The calcium phosphate family of dental materials, including types of cement, restorations, and coatings, is broadly used and well-known for its bioactivity, biocompatibility, and osteoconductive abilities [87]. This family of materials were extensively reported to be utilized in pulp and root canal treatments [88-90], perforations repair [91-93], formation of dentine bridge [94,95], and periapical deformations and bone repair [96].

**Figure 1:**
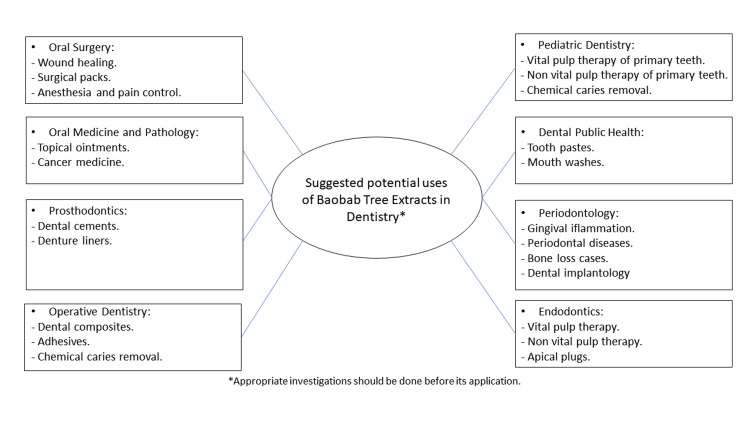
Potential uses of different Baobab tree extracts in dentistry.

Based on the previously discussed components of the Baobab tree and their medical effects, Figure 1 shows suggestions for its applications in dentistry per dental discipline, which are considered areas for further research.

## Conclusions

Under the limitations of this review, the AD tree parts possess bioactive components that have a promising potential to be used in dentistry and dental medicine after proper in vitro investigations. The present review could not find information in the literature regarding using Baobab tree components in dentistry. However, the medical use and pharmaceutical effects of Baobab extracts are evident in the literature. The broad and diverse documented medical and pharmaceutical properties of Baobab tree extracts are mainly correlated to their antioxidant, cell-protective, and anti-inflammatory properties. These effects were attributed to the different tree parts' phytochemical, vitamin C, and phenolic contents. These effects are suggested for further research in multiple areas of the dental field, such as periodontology, endodontology, oral medicine, and dental public health. Furthermore, the high levels of calcium and phosphorus in the tree components could play a role in the regenerative dental biomaterials industry, which demands further studies.
